# The Morphology and Functions of Articular Chondrocytes on a Honeycomb-Patterned Surface

**DOI:** 10.1155/2014/710354

**Published:** 2014-04-06

**Authors:** Joshua O. Eniwumide, Masaru Tanaka, Nobuhiro Nagai, Yuka Morita, Joost de Bruijn, Sadaaki Yamamoto, Shin Onodera, Eiji Kondo, Kazunori Yasuda, Masatsugu Shimomura

**Affiliations:** ^1^Institute of Multidisciplinary Research for Advanced Materials, Tohoku University, 2-1-1. Katahira, Aoba-ku, Sendai 980-8577, Japan; ^2^Creative Research Initiative “Sousei” (CRIS), Hokkaido University, N21 W10 Kita-ku, Sapporo 001-0021, Japan; ^3^Department of Biochemical Engineering, Graduate School of Science and Engineering, Yamagata University, 4-3-16 Jonan, Yonezawa 992-8510, Japan; ^4^School of Engineering and Materials Sciences, Queen Mary University of London, London E1 4NS, UK; ^5^Department of Sports Medicine and Joint Reconstruction Surgery, Hokkaido University School of Medicine, Kita-15 Nishi-7 Kita-ku, Sapporo 060-8638, Japan; ^6^World Premier International Research Center Advanced Institute for Materials Research (WPI-AIMR), Tohoku University, Katahira 2-1-1, Sendai 980-8577, Japan

## Abstract

The present study investigated the potential of a novel micropatterned substrate for neocartilage formation. Articular chondrocytes were cultured on poly(**ɛ**-caprolactone) materials whose surfaces were either flat or honeycomb-patterned. The latter was prepared using a novel self-organization technique, while the former, was prepared by spin-coating. The chondrocytes attached and proliferated on both surfaces. On the honeycomb films, chondrocytes were found at the top surface and encased within the 10 **μ**m pores. Meanwhile, chondrocytes on the spin-coated surface flattened out. Accumulation of DNA and keratin sulphate was comparatively higher on the honeycomb films within the first 7 days. At their respective peaks, DNA concentration increased on the honeycomb and flat surfaces by approximately 210% and 400% of their day 1 values, respectively. However, cultures on the flat surface took longer to peak. Extracellular Matrix (ECM) concentrations peaked at 900% and 320% increases for the honeycomb and flat cultures. Type II collagen was upregulated on the honeycomb and flat surfaces by as much as 28% and 25% of their day 1 values, while aggrecan was downregulated with time, by 3.4% and 7.4%. These initial results demonstrate the potential usefulness of honeycomb-based scaffolds during early cultures neocartilage and soft tissue engineering.

## 1. Introduction

A topical approach to addressing diseased or damaged joint tissues combines cells, often autologous, with either biological or artificial scaffold [1]. The scaffolds function as carriers of the cells [2–5], retaining them at the defect site. In addition to giving structural support and some mechanical integrity, the scaffolds are required to encourage the cells to multiply and produce new ECM. Therefore, the cells must interact with the scaffold, usually by attaching, multiplying, and possibly forming neotissue. In the case of articular cartilage, the cell-material interactions are complicated by the necessity to maintain their rounded morphology [6]. Failure to achieve this may result in their dedifferentiation, changing their pattern of gene expression from chondrocyte-specific to one resembling fibroblastic or chondroprogenitor-like cells. These are associated with the production of type I collagen and nonaggregating glycosaminoglycan (GAG) [7–9]. This morphology and, indeed, the phenotype are successfully maintained by seeding the cells within hydrogels such as agarose and alginate [10, 11]. However, the pore sizes inside these hydrogels may be as little as 2–500 nm for alginate, and up to 500 nm for agarose, depending on their concentrations [12, 13]. In both cases, this results in nutrient deprivation and death of cells at the centre of constructs, thicker than 1 mm [14]. Therefore, there is an ongoing search for ideal scaffold materials, which support chondrocyte proliferation, allow sufficient transport of oxygen and nutrients to cells at all regions and efficient waste removal, maintain their phenotypic functions, and degrade at a rate synchronised with neotissue formation. On this topic, we have reported that honeycomb-patterned porous polymer films (honeycomb films) can be prepared by casting polymers, dissolved in a water-immiscible solvent under high humidity [15, 16]. This technology was adapted for biological applications. Specifically, the morphology and hence the functionality of hepatocytes [17, 18], cardiac myocytes [19], neural progenitor cells [20, 21], and endothelial cells [22] were all manipulated by altering the size and shape of the micropores of the honeycomb films. More recently, bone formation has been found to be enhanced on honeycombed surfaces [23], as well as sustaining the adiponectin secretion by mesenteric-visceral adipocytes over extended culture periods [24]. Chondrocytes have been cultured on several micropatterned surfaces, for an enhanced proliferation compared with nonpatterned surfaces of the same materials [25, 26]. However, maintaining the chondrocytes phenotypes on such surfaces is still a challenge.

In the present study, we observe the behaviour of articular chondrocytes on honeycomb-patterned films. The purposely designed surfaces had highly regular pores, interconnected and 10 *μ*m in diameter. The attachment and changes to their morphology over time were monitored. In addition, the organisation of their cytoskeleton on the honeycomb films was visualised. Their survival and proliferation on the honeycomb films were compared with those on flat nonpatterned substrate derived from the same polymeric materials. Additionally, the accumulation of ECM proteins on the two surfaces was quantified. Finally, we assess the ability of the honeycomb films to maintain the cells phenotype over a prolonged culture period.

## 2. Materials and Methods

### 2.1. Film Preparation

Honeycomb (HC) films were prepared from biodegradable polymers poly(*ɛ*-caprolactone) (PCL; MW = 70,000–100,000) and a copolymer of N-dodecylacrylamide and *ω*-carboxyethyl acrylamide (Cap; MW = 22,000). Cap served as an emulsifier as described in a previous study [15]. Briefly, PCL and Cap (10 : 1 wt%) were dissolved in chloroform at a concentration of 5 g/L. The polymer solution was poured into a round glass dish (9.3 cm in diameter), containing polyethylene terephthalate (PET) discs. The discs, which were 14 mm in diameter, were completely immersed in the polymer solution, while humid air (23% ± 2%) was blown on their surface, at 1.0 L/min. This resulted in films with a 10 *μ*m pore diameter. Alternatively, 0.5 mL of the polymer solution was transferred unto the PET discs dropwise using a pipette. The discs, with the polymer layer, were spun at 1000 rpm for 30 seconds using a spin coater (1H-7D, Mikasa). The resulting specimens contained a flat geometry and hence were termed as* flat films*. These were used as the control groups in comparative studies. Prior to cell culture, both groups of films were immersed in 1-propanol for 12 min to remove the Cap on the surface of the honeycomb films and subsequently sterilised by washing 3 times in pure ethanol and 3 times in sterile, deionised water. This washing procedure was followed by 2-3 hours of exposure to ultraviolet light.

### 2.2. Cell Isolation and Culture

Articular chondrocytes were obtained from 8–10-week Japanese white rabbits. The rabbits, purchased from a local abattoir, were sacrificed using 10 mL injection of 2.5 g/100 mL of Phenobarbiturate (Dainippon, Sumitomo Pharma, Japan). Slices of full thickness cartilage were removed from the rabbits articulating joints, namely, the humeral and femoral heads, and the femoral condyles, using a sterile scalpel, immediately after sacrifice. The explants were kept immersed in a Petri dish containing PBS (Takara Bio, Tokyo, Japan) to maintain hydration. Once all the explants had been removed, the EBSS was aspirated, and the cartilage explants were finely diced using a sterile scalpel. The diced explants were transferred into a 50 mL conical tube, to which 50 *μ*g trypsin dissolved in 10 mL of DMEM supplemented with 10% FCS (Thermo Trace, Australia) solution was added. The falcon tube, containing the explants in trypsin, was incubated in a shaking water-bath at 37°C for 30 minutes. After this time, the trypsin was aspirated and replaced with a solution containing 50 *μ*g of collagenase, dissolved in 50 mL of DMEM (10% FCS). These were incubated in the water-bath at 37°C for 5 hours. The resulting suspension was filtered through a 70 *μ*m sterile cell sieve (BD Biosciences, MA, USA) and centrifuged at 720 g for 5 minutes. The supernatant was aspirated and the cells were washed with DMEM (Invitrogen, Tokyo, Japan) supplemented with 10% FCS (Thermo Trace, Australia) three times. Following this, the cells were expanded in monolayer, by incubation in DMEM, supplemented with 10% FBS at 37°C and 5% CO_2_ for 7 days. The cells were subsequently trypsinised and washed 3 times in serum-supplemented DMEM. After the third wash, the cells were resuspended in 10 mL of DMEM supplemented with 10% FBS (Thermo Trace, Australia) and loaded onto either the flat or honeycomb-patterned films at a concentration of 10 × 10^4^ cells per disc.

The cell-seeded PCL films were transferred to individual wells of 24-well plates (IWAKI, Tokyo, Japan) and cultured in DMEM (supplemented with 10% FCS) for up to 14 days, while samples were obtained at 1, 3, and 7 days.

### 2.3. Scanning Electron Microscopy

Following their culture, samples were dehydrated at 6, 24, and 72 hours by washing in increasing concentrations of ethanol and subsequently dried using a critical point dryer (HCP-2, Hitachi). The dried samples were mounted on aluminium stages, with a double-sided adhesive tape, and coated with a 5 nm layer of palladium gold, using an ion sputter coater (E-1030, Hitachi). These samples were observed using a scanning electron microscope (SEM; S-3500N, Hitachi).

### 2.4. Cytoskeletal Staining and Confocal Laser Scanning Microscopy

Alternatively, samples were transferred to separate multiwell plates. These were then fixed with 3.7% (v/v) formaldehyde for 10 minutes at room temperature and then washed 3 times with PBS. The samples were permeabilised for 15 min with 0.1% Triton X-100 (MP Biomedicals, Eschwege, Germany) and 1.0% bovine serum albumin (Sigma) in PBS. After rinsing twice with PBS, the cells were stained by incubating for 60 minutes with 0.03% (v/v) rhodamine-phalloidin (Molecular Probes, Eugene, OR) in PBS and 6.7% (v/v) of 4′,6-diamidino-2-phenylindole (Dapi; Molecular Probes, Carlsbad, CA) at room temperature. The stained cells were then rinsed and analyzed using the confocal laser scanning microscope (FV-300, Olympus, Tokyo, Japan). The cell nucleus was observed at the excitation and emission wavelengths of 355 nm and 460 nm, respectively, while their actin fibres were observed at the excitation and emission wavelengths of 554 nm and 573 nm, respectively.

### 2.5. DNA

Cultured samples were obtained at predetermined time points. The cells were lysed by vortexing the individual discs in a falcon tube containing 1 mL of a lysate solution, containing 0.5% (v/v) Triton X-100, 150 mM NaCl, and 10 mM HEPES. DNA concentrations within the digested samples were measured using the Quant-Ti, PicoGreen kit (Invitrogen, Tokyo Japan). The commercially available assay, designed to measure double-stranded DNA, was used as directed by the manufacturer's instructions. However briefly, 0.5% (v/v) of the PicoGreen solution was dissolved in the accompanying buffer solution (10 nM Tris-HCL, 1 mM EDTA, pH 7.5). Standard DNA solutions were prepared by mixing Lambda double-stranded DNA in the buffer solution to achieve concentrations of 2000, 200, 20, 2, and 0.2 ng/mL. One hundred microlitres of the standard solutions and the extracted samples were transferred into separate wells of a 96-well plate (Costar, NY, USA) using a pipette. To each well, 100 *μ*L of the PicoGreen solution was added. Fluorescence emission was measured using a commercially available fluorimetry apparatus (TECAN, Tokyo, Japan) at the excitation and emission wavelengths of 485 nm and 535 nm, respectively.

### 2.6. ECM

The samples that were obtained and lysed for DNA measurements were also analyzed for ECM components using the commercially available Keratan Sulphate (KS) [27] ELISA kit (Seikagaku Corporation, Tokyo, Japan). The immunoassay method, which is based on a sandwich of enzymes and a monoclonal antibody specific to KS, was carried out as directed by the manufacturers. However, briefly standard solutions of KS were prepared by diluting the 80 ng/mL of KS with the provided sample diluent to make 40, 20, 10, 5, and 2.5 ng/mL. The sample diluents functioned as blank solution (0 ng/mL). The standard solutions and all reagents and samples were brought to 21 ± 1°C prior to experimentation.

Each well of a 96-well plate (provided) was washed 4 times with 200 *μ*L of the washing solution (provided). Fifty microlitres of the KS standard solutions (and blank) and the experimental samples were transferred into individual wells of the 96-well plate, sealed (with provided cover film), and incubated for 60 minutes at 37°C. The reactants were removed, and each well was washed 4 times with 200 *μ*L of the wash solution. Twenty-five microlitres of Horseradish peroxidase-conjugated streptavidin solution and biotinylated antibody solution were added and mixed gently inside each well. This solution was sealed and incubated for 60 minutes at 37°C. After the incubation period, the reaction solution was removed and each well was washed 4 times with 200 *μ*L of the washing solution. Fifty microlitres of the substrate solution (supplied assay kit) was pipetted into each well, sealed, and incubated in the dark, at 21 ± 1°C. After 10 minutes of incubation, 50 *μ*L of stop solution was added into each well and mixed gently. The absorbance was measured at 450 nm using a microplate reader (Bio-Rad, Tokyo, Japan).

### 2.7. Gene Expression

Total RNA of cultured chondrocytes at each time point was extracted using TRI reagent (Invitrogen, Tokyo, Japan) to evaluate the changes in their gene expression during cell culture. Total RNA was extracted according to the manufacturer's instructions. Yield and purity of the extracted RNA were determined using the spectrophotometer (Smartspec Plus; Bio-Rad, Hercules, CA, USA).

All oligonucleotide primer sets were designed based upon the published mRNA sequence. The expected amplicon lengths ranged from 93 bp to 139 bp. Oligonucleotide primers used in this study are listed in Table 1. Real-time PCR was performed in Thermal Cycler Dice TP800 (Takara, Japan), using SYBR Premix Ex Taq (Takara, Japan). One microliter of cDNA template was used for real-time PCR in a final volume of 25 *μ*L. cDNA was amplified according to the following condition: 95°C for 5 s and 60°C for 30 s at 40 amplification cycles. Changes to their fluorescence were monitored with SYBR Green after every cycle. A dissociation curve analysis was performed (0.5°C/s increase from 60 to 95°C with continuous fluorescence readings) at the end of cycles to ensure that single PCR products were obtained. The amplicon size and reaction specificity were confirmed by 2.5% agarose gel electrophoresis. All reactions were repeated in 4 separate PCR runs, using RNA isolated from 12 samples per time point, for both the honeycomb and flat films. The results were evaluated using the Thermal Cycler Dice Real Time System software program. Glyceroaldehyde-3-phosphate dehydrogenase (GAPDH) primers were used to normalize the samples.

### 2.8. Data Analysis

Numerical data were evaluated by analysis of variance using a commercially available statistical software package. A statistical significance was deduced when the *P* value is less than 0.05.

## 3. Results

### 3.1. Cell Attachment

**Figure 1:**
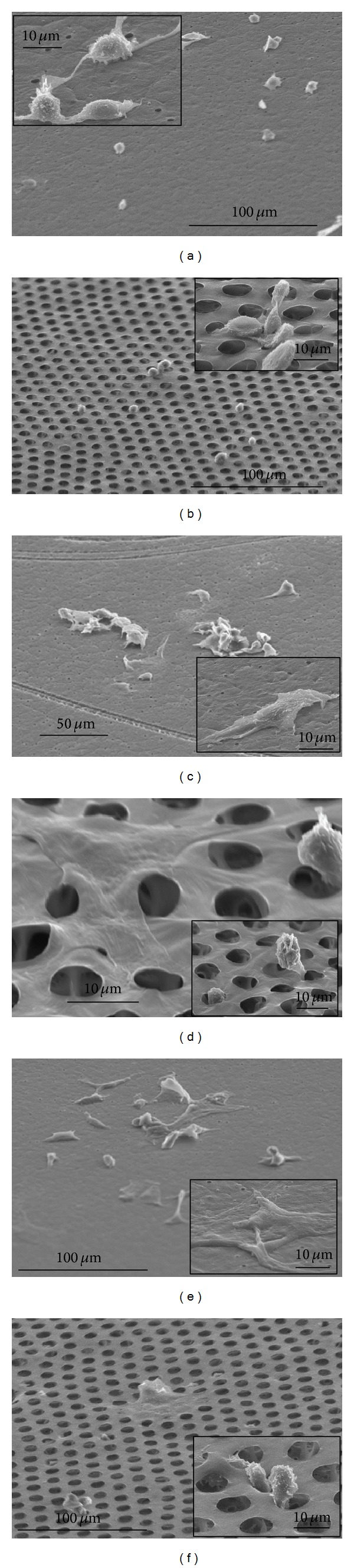
Representative scanning electron micrographs of articular chondrocytes, cultured on flat ((a), (c), and (e)) and honeycomb films ((b), (d), and (f)) for 6 ((a), (b)), 24 ((c), (d)), and 72 ((e), (f)) hours.

Early morphological changes to the chondrocytes on both the flat and honeycomb films were observed using scanning electron microscopy. The micrographs in Figures 1(a) and 1(b) show the distribution and morphology of chondrocytes after 6 hours of culture on both the flat and HC films. Observably, cells on both films were morphologically rounded. While the cells demonstrated very little spreading, some cell-cell interactions were observed by those within close proximity on the flat film. Cells on the HC, however, either had processes which extended inside or were physically encapsulated within the pores. By 24 hours, more cells may be observed on the films. Although not completely flattened, some of the cells on the flat film had begun adopting a different morphology (Figure 1(c)), taking over a larger area on the films. At the same time, chondrocytes on the honeycomb films were either completely flattened, covering areas of several pore sizes, or still rounded, with processes inside the pores (Figure 1(d)). At 72 hours, cells cultured on the flat films (Figure 1(c)) appeared relatively flatter than earlier time points observed. However, some cells, particularly on the honeycomb films, maintained their rounded morphology. Despite this, chondrocytes had begun to spread over the honeycomb films, covering several adjacent micropores (Figure 1(f)).

### 3.2. Cytoskeletal Organisation

**Figure 2:**
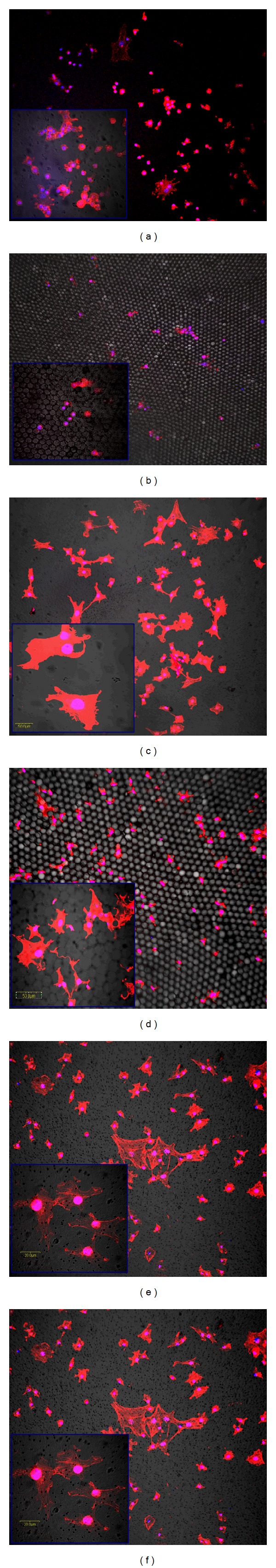
Low and high magnification confocal images of chondrocytes whose nuclei and cytoskeletal fibres were stained following 6 ((a), (b)), 24 ((c), (d)), and 72 ((e), (f)) hours of culture on either flat ((a), (c), and (e)) or honeycomb ((b), (d), and (f)) films.

The dual confocal images in Figure 2 show the cell nucleus (blue) and their cytoskeletal actin fibres (red) following 6, 24, and 72 hours of culture. Collectively, these confocal images suggest an increased cell number over the 72-hour period. Additionally, the increasing surface area taken up by the cells actin network is indicative of their spreading. At 6 hours, the area covered by the actin fibres on either the flat or patterned films was not much bigger than that of the cell nucleus (Figures 2(a) and 2(b)). There were some exceptions, whereby, individual cells had produced and organised a relatively high amount of actin fibres. However, these were only associated with cells on the flat films. The micrographs corresponding to 24 hours showed that most of the cells were beginning to develop their cytoskeleton. However, greater extent of actin formation was observed by cells on the flat film (Figure 2(c)), compared with those on the honeycomb films (Figure 2(d)). In addition, the cells on the flat films had begun to form small clusters, consisting of 4–6 cells. This trend continued till 72 hours, where most cells on the flat films (Figure 2(e)) exhibited a relatively well-organised cytoskeleton compared to those on the honeycomb films (Figure 2(f)). Despite cell spreading being highest at 72 hours, a large proportion of cells still had less pronounced actin fibres. Moreover, the size differences between their nuclei and their network of actin fibres were not significantly different from those of cells imaged after 6 hours of culture. These cells were more prevalent on the honeycomb films.

### 3.3. DNA and GAG Concentrations

**Figure 3:**
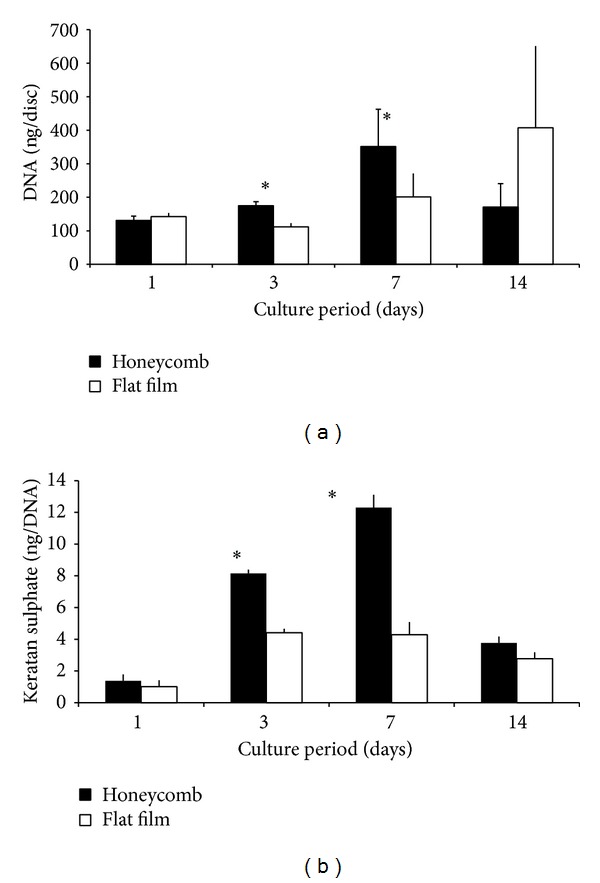
Graphs show (a) DNA and (b) keratin sulphate concentrations measured on honeycomb and flat films over the 14-day culture period. Data represents mean and standard deviations of 3–6 discs per sampled time point *P < 0.05.

The concentrations of DNA and keratan sulphate retained on the films are summarised in Figure 3. For both the flat and HC films, the DNA concentration increased steadily over the 14 days. The peak DNA increase on the HC films occurred on day 7. The value was 200% of the day 1 value. Thereafter, DNA was reduced at day 14. By contrast, DNA concentration on the flat films peaked on day 14, with a 400% increase from the day 1 value.

Figure 3(b) shows keratan sulphate retained on the films, normalised to DNA concentration, over the 14-day culture period. The graph demonstrated steady increases with culture time. Evidently, keratin sulphate concentrations were higher on the honeycomb films at every time point than on those associated with the flat films. Accordingly, the biggest and most significant differences were at days 3 and 7. Keratin sulphate values for the HC and flat films peaked on day 7, after which they reduced from 12 and 6 ng/disk, respectively, to 4 and 3 ng/disk, which represents approximately 400% and 300% of their respective day 1 values.

### 3.4. Gene Expression

**Figure 4:**
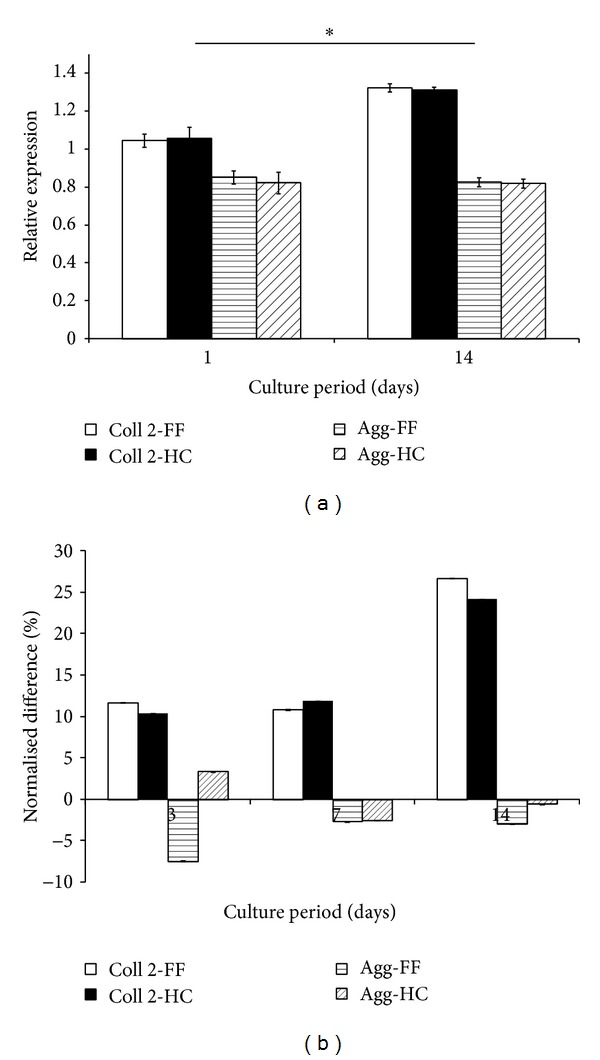
Graphs show (a) the expressions (relative to that of GAPDH) of collagen type 2 (Coll 2) and Aggrecan (Agg) after 1 and 14 days of culture on either the honeycomb (CH) or the flat (FF) films. Normalised to their day 1 values, (b) shows the temporal changes to their expression levels at days 3, 7, and 14, in terms of percentage difference. Data represents mean and standard deviations of 12 discs per sampled time point.

The expression of mRNA for aggrecan and type II collagen, relative to GAPDH, are presented in Figure 4. It is clearly demonstrated that the relative expressions of neither the type II collagen nor the aggrecan differed between the flat and honeycomb films. Accordingly, aggrecan expression was always lower than that of type II collagen, irrespective of surface topography. Furthermore, the expression of type II collagen increased over the 14 days, while that of aggrecan appeared to have decreased slightly (ns).  Figure 4(b) describes the temporal changes to these relative expressions at 3, 7, and 14 days. These were obtained by normalizing the corresponding time points to day 1 values to obtain the percentage differences. It may be observed that there was approximately 10% upregulation of type II collage on both films, at days 3 and 7. By 14 days, this rose to approximately 25% of the day 1 values, with insignificant differences between cells on the honeycomb films and the flat films. By contrast, expression for aggrecan by cells on flat film was downregulated over the culture period. A peak downregulation of 7.4% was observed at day 3. However, by day 14, the reduction had reduced to approximately 2.9% of their day 1 values. With regard to cells on honeycomb films, there was an initial upregulation of 3.4%. However, by 14 days, aggrecan expression had reduced only by a mere 0.6% of its day 1 value.

**Figure 5:**
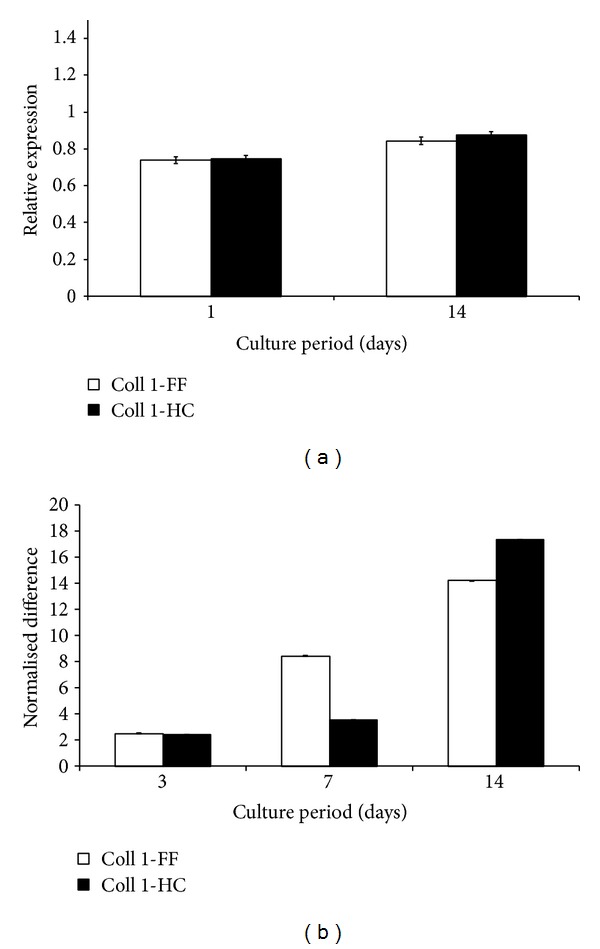
Graphs show (a) the expression (relative to that of GAPDH) of collagen type 1 (Coll 1) after 1 and 14 days of culture on either the honeycomb (CH) or the flat (FF) films. Normalised to its day 1 values, (b) shows the temporal changes to the expression levels at days 3, 7, and 14, in terms of percentage difference. Data represents mean and standard deviations of 12 discs per sampled time point.

The graphs in Figure 5 show the relative expression of type I collagen and the temporal changes to its expression on both films. A significant level of type I collagen, relative to GAPDH, was achieved on both films, from as early as day 1. Although not surpassing GAPDH, the expression for type I collagen increased steadily over the remaining culture period. The up-regulation of type I collagen increased from approximately 2.5% at day 3 on both film, to 14% and 17% on the flat and honeycomb films, respectively, at day 14. Interestingly, the upregulation type I collagen was higher on flat films than honeycomb at day 7, with their normalised values being 8.4% and 3.6%, respectively.

## 4. Discussion

The present study examined the behaviour of articular chondrocytes on HC films prepared using a novel self-organization method. At first, it was useful to determine whether the micropatterned surface was suitable for the chondrocytes, since their morphology is important and is governed* in vitro* by their spatial environment. Cytotoxicity on PCL surfaces was not a major concern, since porcine articular chondrocytes have been successfully cultured on PCL for up to 14 days by Tsai and colleagues [28]. Moreover, Cap, which served as an emulsifier has been used to make honeycomb-patterned films, and hepatocytes were successfully cultured [18].

The chondrocytes attached to both the flat and HC films PCL films used in the present study, the articular chondrocytes attached to both the flat and the honeycombed PCL surfaces. At the earliest observed time point, the cells on both surfaces were typically round, sparse, and heterogeneously distributed. Although not quantitatively compared, the honeycomb films retained more cells at 6 hours than the flat films. SEM micrographs showed some cells within the pores. These cells were morphologically rounded, suggesting that they were physically entrapped.

The 24-hour samples had significantly higher cell number and were more homogeneously distributed on both films. It is, however, unlikely that proliferation is responsible for this. Rather, it is more plausible that the chondrocytes adhesion to the films at 6 hours were not strong enough to survive the preparation process required for SEM imaging, resulting in a significant amount of the cell detachment. Between 6 and 24 hours more cells had attached to the surfaces, and stronger interactions had been formed. Presumably, this reduced cell detachment during the SEM sample preparation.

The organisation of chondrocytes actin cytoskeleton has been shown to correlate with their differentiation and gene expression [29]. Therefore, monitoring its developments on the microporous films was potentially useful for predicting their midterm survival and long-term functionality. The flattened appearance of the articular chondrocytes at 72 hours, combined with the larger surface area covered by their actin fibres, at the same time point, suggests that the majority of the cells had begun to spread on the films. Prior to this, their cytoskeletal organisation exhibited a ring-like distribution. This ring-like distribution was also observed in native cartilage by Ciolfi and coworkers [30], who suggested this to be their preferred actin organisation. By contrast, the actin filaments observed at 72 hours were extensive and appeared randomly organised, with respect to the honeycomb films.

The temporal changes to DNA concentration described the cells proliferation over the 14 days. For cells cultured on flat films, the biggest difference in DNA values occurring between days 7 and 14 suggests that cellular proliferation on the flat films was highest then. On the other hand, cell proliferation on the HC films peaked between days and 3 and 7. The reduced DNA concentration may either be an indication of a pending viability issue or an incomplete digestion of the day 14 HC samples. Due to the heterogeneity of the HC-cellular constructs, it is likely that, during the course of time, the cells at the top flatten out and produce ECM. The ECM inevitably hinders access to the internal regions of the HC pores. A natural consequence is that the cells within may suffer from reduced nutrient supply. Indeed, cases of nutrient deficiency leading to reduced cellular activities and viabilities within 3D-cultures are all too common in the literatures. A further consequence is that only the cells (flattened) at the top of the HC surfaces, along with their ECM, were digested sufficiently to allow detection by the biochemical reagents. This is a probable explanation for the lower values of ECM, GAG and gene expression molecules measured from HC cultures.

Cellular viability was monitored regularly during the study using Calcein-AM-Ethidium Homodimer method. There was no viability issue observed with either of the culture setups. Therefore, it is fair to assume that the lower experimental parameters observed with the HC-cultures are most likely due to the incomplete digestion of the 3D-samples, caused by lack of access to the internal regions of the micropores, as opposed to a cellular viability challenge.

Interestingly, the amount of DNA associated with the flat films at day 14 is not significantly different to that on honeycomb at day 7. This implies either that cell proliferation on flat film has a longer lag phase than when cultured on the honeycomb films, or that this DNA value represents a maximum number of cells sustainable by the 14 mm diameter discs. The latter explanation is however unlikely, since cells are capable of forming multiple layers on flat and microporous surfaces [31]. By extending the culture period, it may be possible to determine the true maximum DNA value, that is to say, the maximum number of cells sustainable by both films and how long it takes for the films to achieve their respective values.

The increased keratin sulphate concentration over the 14 days suggested that the cells on both films were producing ECM. Observably, ECM concentration on both types of surfaces peaked at day 7 and fell significantly thereafter, while the decline on the flat surface during the same time point was not significant. Matrix production by human chondrocytes, cultured on both flat and porous PCL scaffolds were previously observed to increase till 14 days, and decline henceforth [32].

While the keratin sulphate concentration on the films provides good indication of its production by the cells, it is worth noting that the higher concentration associated with HC films may result also from a higher retention on the microporous surface. Indeed, it is well reported that surface topography influences protein adsorption [33]. Moreover, adsorption of fibronectin and vitronectin to honeycomb films was found to be significantly higher than that on the corresponding flat surface [34]. This is conceivable, since the surface area of honeycomb films is higher than that of flat films [35]. Keratin sulphate is a small glycosaminoglycan (GAG) unit, essential to the formation of the larger, aggregating proteoglycan, aggrecan. As GAGs build up, aggrecan molecules are formed. These bind noncovalently to hyaluronan chains, to form an aggrecan-hyaluronan complex. This aggregating GAG molecule, whose chain length and molecular weight may reach 10,000 nm and 50,000 kDa, respectively, is involved in tissue hydration, load distribution, and the immobilisation and storage of growth factors [36]. Therefore, the comparatively high retention of keratin sulphate on the HC films may indicate a superior ability to induce neocartilage with functional ECM.

In a similar study, [26], chondrocytes were found to survive on polymeric surfaces with 5 *μ*m pore. Their proliferation and matrix synthesis were validated for up to 3 weeks. However, the cells were observed to spread on the films, adopting a flat morphology. Moreover, it was not determined whether the surfaces had maintained the cells phenotype. Notably, the 5 *μ*m pores are insufficient to accommodate the chondrocytes, therefore, excluding them from the internal surfaces. The present study assessed the possibility of maintaining the viability and morphology of articular chondrocytes on HC-patterned surfaces with 10 *μ*m pore diameter. Quantifying RT-PCR helped determine the gene expression levels of type II collagen and aggrecan. These genes had been identified as the major phenotypic markers for chondrogenic tissues [37–40]. Their levels of expression were compared to that of the protein, glyceraldehyde-3-phosphate dehydrogenase (GAPDH). The upregulation of type II collagen expression at days 3 and 7 (Figure 4(b)) correlates with the increased matrix synthesis (Figure 3(b)) observed at these time points. Furthermore, as the accumulation of matrix molecules on the cultured discs fell at day 14, the expression of collagen II, the major component of chondrocytes ECM, increased. This response may be associated with matrix turnover, during which anabolic and catabolic gene expression compete to balance matrix homeostasis [41]. On the other hand, the temporal downregulation of aggrecan (Figure 4(b)) may suggest that the chondrocytes were undergoing some sort of phenotypic change. The heterogeneity of chondrocytes within cartilage is such that the superficial zone of articular cartilage characteristically has one to several layers of flattened, disc-shaped cells. As well as having densely packed bundles of collagen fibres orientated parallel to the articulating surface, these cells are associated with relatively low proteoglycan content [36, 42]. Bearing in mind the fact that the chondrocytes had begun to spread over the films, adopting a flattened morphology by 72 hours (Figures 1(e) and 1(f)), it is therefore plausible that the cells were developing characteristics associated with cells at the superficial zones of articular cartilage. The chondrocytes situated inside the 10 *μ*m pores typically maintained their rounded morphology (Figures 1(d) and 1(f)). This may explain why the downregulation of aggrecan was less pronounced by cells on the HC films, compared to those cultured on the flat films, which had completely flattened out.

The expression level for type I collagen was measured to detect dedifferentiation of the chondrocytes back into their more fibroblastic progenitors. At 24 hours expression of type I collagen, relative to GAPDH, was approximately 0.74, compared to 1.04 and 0.85 of type II collagen and aggrecan, respectively. Nonetheless, expression of type I collagen was unexpected. It is worth noting that prior to their culture on the flat and honeycomb films, the cells were expanded in monolayer for 7days. Such culture has been reported to induce chondrocytes dedifferentiation and the production of type I collagen [10, 36]. It is therefore likely that the expression of type I collagen by the cells from as early as 24 hours may have resulted from their expansion in monolayer. Moreover, the temporal upregulation of the gene for type I collagen suggests a continual trend by some cells to dedifferentiate, despite the simultaneous upregulation of the type II collagen gene.

## 5. Conclusions

In light of the observations made in this study, it is concluded that articular chondrocytes attach to honeycomb surfaces. This was manifested by increased concentration of DNA and accumulation of extracellular matrix molecules. The extent of these was either similar to or, at times, greater than the flat surfaces, within the first 7 days. However, by 14 days, the honeycomb fell short of the flat films.

Chondrocytes, being naturally round, may have been adversely affected by their adoption of a flat morphology when attached to and spread on the flat and honeycomb films. The extent of this was difficult to determine, since the onset of phenotypic change may have begun during the preculture phase.

Extending the culture period may be useful to observe the long-term fate of cells on the honeycomb surfaces. It may be possible that the formation of multilayer of cells, albeit heterogeneous in morphology, with the rounded cells within the pores, while the flattened ones are at the top, may produce neocartilage, which is structurally and functionally similar to articular cartilage. Furthermore, by manipulating the pore geometry of the honeycomb films, the advantage achieved, in the first 7 days, with respect to both proliferation and matrix synthesis, may be prolonged.

## Figures and Tables

**Table 1:** List of primers used in real-time PCR.

Primer ID	Primers (5′-3′)	Expect size (bp)	Accession number
Collagen II-F Collagen II-R	GAC CATC AAT GGC GGC TTC CAC GCT GTT CTT GCA GTG GTAG	139	D83228.1

Aggrecan-F Aggrecan-R	GCT ACG ACG CCA TCT GCT AC GTC TGG ACC GTG ATG TCC TC	94	L38480.1

Collagen I-F Collagen I-R	GTT CTC AGG GTA GCC AAG GTC AGT CTC CAT CAT AAC CAA AGT CGT A	105	D49399.1

GAPDH-F GAPDH-R	CCC TCA ATG ACC ACT TTG TGA A AGG CCA TGT GGA CCA TGAG	93	L23961.1
